# Using Mobile Phones to Collect Patient Data: Lessons Learned From the SIMPle Study

**DOI:** 10.2196/resprot.6389

**Published:** 2017-04-25

**Authors:** Sinead Duane, Meera Tandan, Andrew W Murphy, Akke Vellinga

**Affiliations:** ^1^ Discipline of General Practice National University of Ireland Galway Galway Ireland

**Keywords:** mobile phone apps, mobile survey, antimicrobial resistance, primary care, quantitative, prescribing, urinary tract infection

## Abstract

**Background:**

Mobile phones offer new opportunities to efficiently and interactively collect real-time data from patients with acute illnesses, such as urinary tract infections (UTIs). One of the main benefits of using mobile data collection methods is automated data upload, which can reduce the chance of data loss, an issue when using other data collection methods such as paper-based surveys.

**Objective:**

The aim was to explore differences in collecting data from patients with UTI using text messaging, a mobile phone app (UTI diary), and an online survey. This paper provides lessons learned from integrating mobile data collection into a randomized controlled trial.

**Methods:**

Participants included UTI patients consulting in general practices that were participating in the Supporting the Improvement and Management of UTI (SIMPle) study. SIMPle was designed to improve prescribing antimicrobial therapies for UTI in the community. Patients were invited to reply to questions regarding their UTI either via a prospective text message survey, a mobile phone app (UTI diary), or a retrospective online survey. Data were collected from 329 patients who opted in to the text message survey, 71 UTI patients through the mobile phone UTI symptom diary app, and 91 online survey participants.

**Results:**

The age profile of UTI diary app users was younger than that of the text message and online survey users. The largest dropout for both the text message survey respondents and UTI diary app users was after the initial opt-in message; once the participants completed question 1 of the text message survey or day 2 in the UTI diary app, they were more likely to respond to the remaining questions/days.

**Conclusions:**

This feasibility study highlights the potential of using mobile data collection methods to capture patient data. As well as improving the efficiency of data collection, these novel approaches highlight the advantage of collecting data in real time across multiple time points. There was little variation in the number of patients responding between text message survey, UTI diary, and online survey, but more patients participated in the text message survey than the UTI diary app. The choice between designing a text message survey or UTI diary app will depend on the age profile of patients and the type of information the researchers’ desire.

**Trial Registration:**

ClinicalTrials.gov NCT01913860; https://clinicaltrials.gov/ct2/show/NCT01913860 (Archived by WebCite at http://www.webcitation.org/6pfgCztgT).

## Introduction

Paper-based surveys have been the standard for collecting patient data in health research. This data collection method is limited due to issues related to data entry and storage costs. Mobile phones offer new opportunities to collect real-time data in a much more efficient and interactive way including automated data upload without data loss, which can be an issue when using other data collection methods [1]. Mobile phones have already been used successfully in the past in the development of health and behavioral change interventions. Examples include in the areas of diabetes self-management, weight loss, physical activity, smoking cessation, and medication adherence [2].

Text messaging or short message service (SMS) has become a ubiquitous method of communication displacing more traditional landline infrastructures [3]. In 2009, Irish citizens were the second-highest users of SMS text messaging in Europe, sending an average of 2700 text messages per year [4]. In 2010, there were an estimated 5.5 million mobile phone subscriptions in Ireland equating to a mobile phone penetration of 119% [5]. Text messaging is fast and convenient giving users flexibility to respond at any time or place and presenting new opportunities to evaluate health-related interventions. The use of automated text messaging services for evaluating health interventions is growing.

According to a recent survey, 75% of people in Ireland are mobile phone users [6]. In 2009, worldwide mobile app downloads amounted to approximately 2.52 billion and are expected to reach 268.69 billion in 2017 [7]. Mobile phones offer researchers new data collection opportunities due to the way in which they are used and how data are shared. Mobile phone apps can reduce data management and processing time for researchers; however, technical difficulties are considered a disadvantage [8]. Mobile phones have been used in the self-management of health and as an adaptive learning, sharing, and social support platform for individuals [9]. However, only recently have apps been used to capture data related to health for the purposes of scientific research. One of the first initiatives in this direction has recently been launched by Apple, who introduced a mobile platform for biomedical research to boost large-scale health studies [10]. Mobile apps are transforming how medicine is conducted and taught in the health care setting [11]; however, there is little evidence of how they can be used to rigorously monitor patient outcomes. To our knowledge, no study has captured real-time data from patients to record urinary tract infection (UTI) symptoms and treatment using a mobile phone app.

This is the also the first time SMS text messaging and mobile phone apps have been used as part of an evaluation of a complex intervention. This paper explores the feasibility of using novel mobile data collection methods to enhance evaluation of complex interventions. This paper illustrates how patient data were collected via a text messaging survey, mobile phone symptom diary app, and retrospective online questionnaire within the Supporting the Improvement and Management of UTI (SIMPle) study. It concludes by discussing the lessons learned from adopting these novel approaches and the potential implications.

## Methods

### Procedure

**Figure 1:**
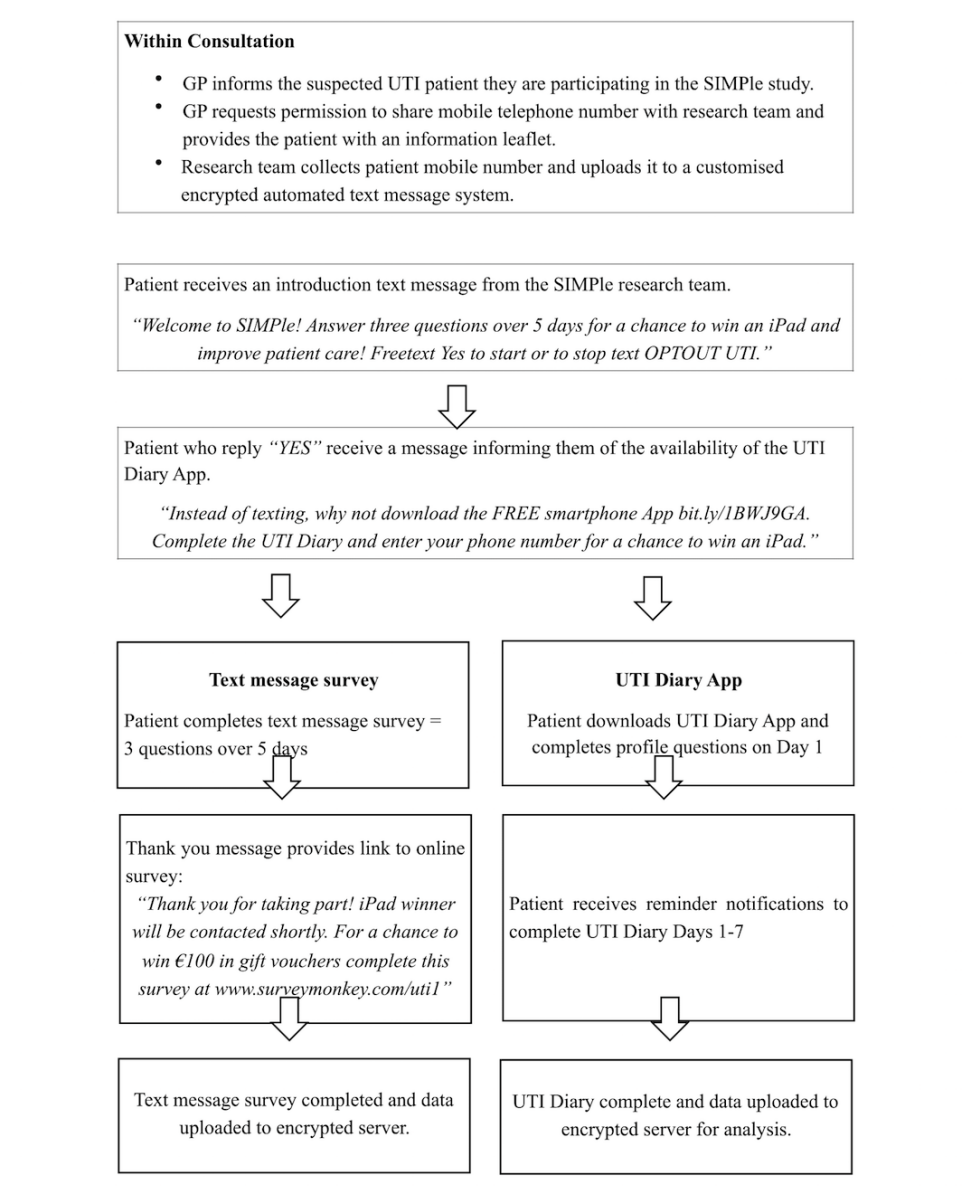
Data collection procedures for the SIMPIe study.

Data were collected from UTI patients through text message survey, a mobile phone symptom diary app (UTI diary), and an online survey. UTIs are the second-most common infections presenting in primary care. Symptoms include feeling unwell, frequency and urgency of urination, pain when passing urine, and pain in the lower abdomen [12]. General practitioners (GPs) from 30 practices participating in the SIMPle study [13] were asked to invite patients with suspected UTI to provide their mobile phone number to the research team. Figure 1 summarizes how the data were collected. Ethical approval was granted for this study through the Irish College of General Practitioners research ethics committee.

The research team initiated contact with UTI patients via text message. The first text message confirmed consent before further participation. Patients who replied “yes” (indicating consent) were invited to complete a text message survey or download the UTI diary app. Patients who completed the text message survey were sent a link to an online survey once they had responded to three questions over five days.

### Text Message Survey

The text message survey was designed to capture data from patients on the type of treatment they recieved, when they started antimicrobial treatment (if at all), and the duration of symptoms. The text message workflow was designed using a customized process that included a 24-hour delay between each question. Text messages were sent at noon each day. Each question used a different keyword (yes, UTI, start, day, and optout UTI). These keywords were used to differentiate responses to each of the questions. Questions were limited to 160 characters including spaces, keywords, response options, and opt-out instructions. An example of one of the questions was: *“* Did the GP give you a) antibiotic prescription b) antibiotic prescription & asked to wait 2 days c) other. Freetext UTI & the answer (eg UTI a) or OPTOUT UTI.” All messages were free to send and receive and patients could opt out of the process at any stage. Text messages were pretested for comprehension.

### Urinary Tract Infection Diary App

The UTI diary app and online survey focused on examining the type of symptoms, severity of symptoms, and treatment recommended. Questions were developed from previous international qualitative and quantitative studies and further expert opinion. Both the UTI diary app and online survey were pretested to ensure face validity of measures and usability.

**Figure 2:**
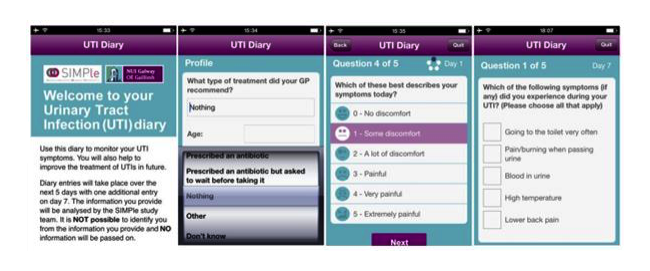
Screenshots of the UTI diary app.

The UTI diary app captured data in “real time” over 7 days (days 1-5 and day 7; Figure 2). The UTI diary app was compatible with Android and iOS (Apple) platforms. On downloading the UTI diary app (day 1), participants completed profile questions (age, gender, employment status), general health, severity of symptoms, and outlined the type of treatment the GP recommended. On days 2 to 5, the same two questions were repeated: severity of symptoms (pain scale) and medication taken (if any). On day 7, participants were asked the same questions as days 2 to 5 with three additional questions on symptoms, satisfaction of information provided at the GP consultation, and general health status. The UTI diary app participants received daily reminders to complete their diary entry.

Participants who completed the text message survey and UTI diary were entered into a draw to win an iPad.

### Online Survey

The online survey was completed approximately 5 days after the UTI consultation via SurveyMonkey. The online survey was much longer than the UTI diary and included 23 questions on patient satisfaction with the consultation, type and severity of symptoms, treatment, and demographics. The online survey included more extensive scales on patient satisfaction and patient demographics, for example. Online survey participants were entered into a draw to win €120 of vouchers.

### Data Management and Analysis

Data from the text message survey and UTI diary app were remotely uploaded and transferred to a secure password-encrypted database. Online survey data were downloaded from the SurveyMonkey database. Missing data were coded prior to analysis. The text message survey, UTI diary app, and online survey were analyzed separately. The data were analyzed to describe participants’ demographic characteristics and symptoms and severity experienced. All participants’ answers were automatically entered into a data file, which was checked for accuracy by two independent researchers.

Data are presented as frequencies and univariate analysis was performed using chi-square tests (at *P* ≤.05, 95% confidence interval) to identify variables associated with antimicrobial prescribing among UTI diary app users only. To evaluate if antimicrobials improved the speed of recovery, a variable was created to indicate the day on which they were considered improved (set at level 2 or less on the pain scale and a second analysis was performed with level 3 or less). Based on this outcome, a Cox proportional hazards model with antimicrobial prescription as an independent predictor was calculated. Data were analyzed using SPSS version 21.0.

## Results

### Participants and Sample

**Figure 3:**
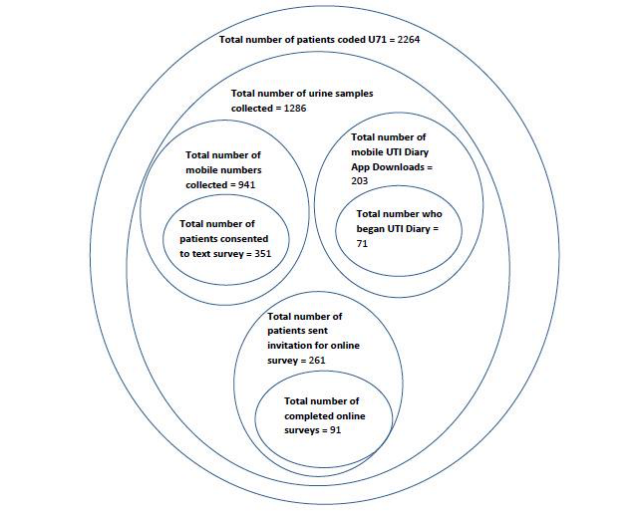
Summary of sampling frame.

During the SIMPle intervention period (9 months), 2264 patients were coded U71 in the GP patient management software, meaning these patients were identified as patients with UTIs [14]. GPs were asked to submit a urine sample to the laboratory for all patients who they coded as U71; patient mobile phone numbers were written on the urine sample form and collected by the researchers. During the intervention period, a urine sample was obtained and sent to the laboratory from 1286 patients or approximately 50% of index consultations. A total of 941 mobile phone numbers were collected from these urine sample forms and these patients were sent an invitation text message to participate in the text message survey. Of these, 351 (37.3%) patients responded to the initial invitation to participate in the text message survey. Twenty-two participants were excluded from analysis due to missing data; therefore, a total of 329 participants answered question 1 of the text message survey. The UTI diary app was downloaded 203 times (175 iOS users and 28 Android users) over a 6-month period. Of participants who downloaded the UTI diary app, 71 (35.0%) responded of whom 31 completed the 7 days of the UTI diary app. Of the 261 who completed the text message survey, 91 (34.9%) responded to the online survey (Figure 3).

**Table 1:** Demographic characteristics of participants (N=491).

Characteristics		Text message survey, n (%) (n=329)	UTI diary app, n (%) (n=71)	Online survey, n (%) (n=91)
**Age (years)**				
	18-24	34 (10.3)	30 (42)	13 (14)
	25-34	62 (18.8)	18 (25)	23 (25)
	35-44	63 (1901)	13 (18)	28 (31)
	45-54	64 (19.5)	6 (9)	13 (14)
	>55	106 (32)	4 (6)	14 (15)
**Gender**				
	Male	22 (6.3)	12 (17)	4 (4)
	Female	326 (91.7)	59 (83)	87 (96)
	Unknown	7 (2)		
**Employment**				
	Employed	—	50 (70)	65 (71)
	Unemployed	—	10 (14)	15 (17)
	Students	—	11 (16)	11 (12)
**Antimicrobials prescribed^a^**				
	Yes	132 (88)	51 (72)	86 (95)
	No	18 (12)	20 (28)	5 (5)

^a^ For text message survey, n=150.

### Demographic Characteristics

Demographic characteristics of the text message survey, UTI diary app, and online survey participants are provided in Table 1.

Across the three data collection tools, participants were mostly female. The majority of UTI diary app participants were aged between 18 and 34 years (67%, 48/71). This was a much younger age profile than those completing the text message survey (28.6%, 94/329) and online participants (40%, 36/91) for the same age group. Those older than 35 years represented 32% (23/71) in UTI diary app group, 71.1% (234/329) of the text message group, and 60% (55/91) of the online survey group.

The majority of participants received an antimicrobial prescription and this was similar in the UTI diary app group (72%, 51/71) and text messaging group (88.8%, 132/150), but much higher in the online survey group (95%, 86/91).

### Text Message Survey Response

The time taken to respond to the text survey varied between participants. For the opt-in message and question 1, more than half of participants responded in less than 1 hour, whereas a further 30% took more than 12 hours to respond. For questions 2 and 3, nearly all participants responded within less than an hour (Q2: 97.5%, 196/201; Q3: 99.5%, 213/214).

Table 2 summarizes the number of times each message was sent before a response was received. The largest dropout from respondents was after the initial opt-in message; once the participant completed question 1, they were more likely to respond to the remaining questions.

The majority of participants who choose to opt out (22.8%, 99/351) did so at the beginning of the process. Nearly 24% did not respond to questions 2 (63/269) and 3 (63/265).

Participants sometimes used incorrect keywords, such as “UTI” instead of the question 2 keyword “start.” When wrong keywords were used, the responses were removed from the analysis.

**Table 2:** Number of times the text message questions were sent to UTI patients.

Number of times message sent	Opt-in message, n (%) (n=351)	Question 1, n (%) (n=270)	Question 2, n (%) (n=268)	Question 3, n (%) (n=263)	Thank you message, n (%) (n=261)
1	251 (71.5)	154 (57.0)	161 (60.1)	190 (72.2)	261 (100)
2	99 (28.2)	116 (43.0)	100 (37.3)	73 (27.8)	—
3	1 (0.3)	—	4 (1.5)	—	—
4	—	—	2 (0.7)	—	—
5	—	—	1 (0.4)	—	—

### Urinary Tract Infection Diary App

Unlike the text message survey, there was no pattern to when people completed the UTI diary. The participants’ response times depended on when they downloaded the UTI diary app.

Table 3 summarizes the overall responses for the UTI diary app. Similar to the text message survey, there was a drop off between opting in on day 1 and days 2 to 7. However, Table 3 highlights that once participants completed day 2 they were less likely to drop out of participating in the UTI diary app. Finally, Table 3 also highlights that UTI diary participants did not skip any questions when completing the UTI diary app; therefore, all fields provided the researchers with data.

Despite the relatively low number of responses to the UTI diary app, the potential of this feasibility study is demonstrated in the analysis of the answers.

### Urinary Tract Infection Diary Response

#### Severity of Symptoms

Table 4 compares severity of symptoms reported on day 1 through the UTI diary with the retrospective account of symptoms on day 5 from the online survey. Overall, double the online survey participants (39%, 35/91) retrospectively rated their symptoms to be severe compared to 18% (13/71) of participants providing real-time data through the UTI diary app.

**Table 3:** Overall number of participants responding to each question in the UTI diary app.

App questions			Day, n (%)										
			1 (n=71)		2 (n=71)		3 (n=46)		4 (n=42)		5 (n=38)		7 (n=33)
**Profile questions**													
	What type of treatment did your GP recommend?	71 (100)		—		—		—		—		—	
	Gender	71 (100)		—		—		—		—		—	
	Age	71 (100)		—		—		—		—		—	
	Number of children	71 (100)		—		—		—		—		—	
	Work situations	71 (100)		—		—		—		—		—	
**Health questions**													
	How good or bad is your health today?	71 (100)		—		—		—		—		—	
	How is your health in general?	71 (100)		—		—		—		—		—	
	Overall, how satisfied were you with the treatment recommended by your GP?	71 (100)		—		—		—		—		—	
	Which of these best describes your symptoms today?	71 (100)		46 (65)		42 (91)		38 (91)		33 (87)		—	
	What medication have you taken today?	71 (100)		46 (65)		42 (91)		38 (91)		33 (87)		—	
	Which of the following symptoms (if any) did you experience during your UTI?	—		—		—		—		—		31 (94)	
	How good or bad is your health today?	—		—		—		—		—		31 (94)	
	How is your health in general?	—		—		—		—		—		31 (94)	
	Overall, how satisfied were you with the treatment recommended by your GP?	—		—		—		—		—		31 (94)	
	How good was your GP at explaining your treatment for your UTI?	—		—		—		—		—		31 (94)	

**Table 4:** Severity of symptoms for real-time participants (app) compared to retrospective participants (online survey).

UTI symptoms rated	UTI diary app, n (%) (n=71)	Online survey, n (%) (n=91)
Mild	28 (39)	6 (7)
Moderate	30 (42)	50 (55)
Severe	13 (18)	35 (39)

Among UTI diary app participants, a significant decrease in severity was observed between day 1 and day 2 (χ^2^_1_=5.2, *P*=.02; Table 5). Of the participants who indicated worsening of symptoms between days 1 and 2, 64% (21/33) started antimicrobial therapy immediately.

**Figure 4:**
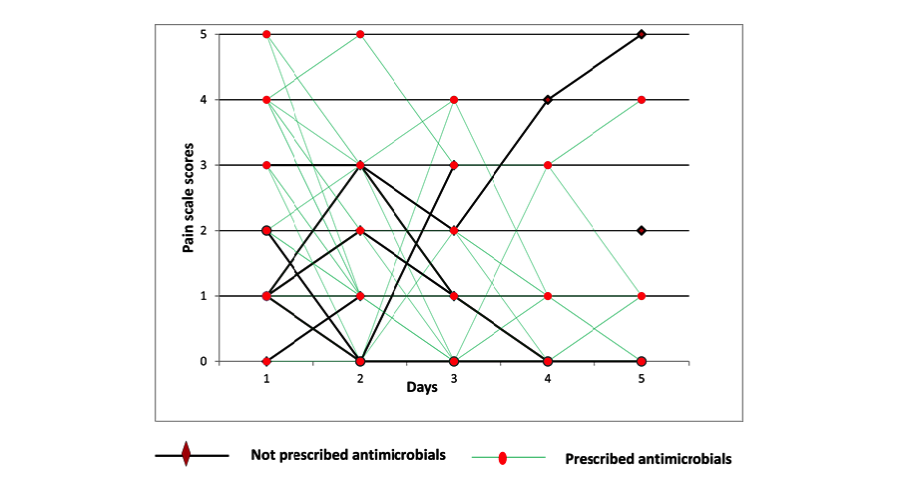
Severity symptoms rated by apps participants from days 1 to 5 (n=71).

Figure 4 and Table 6 illustrate the severity of symptoms rated by UTI diary app users over 5 days (days 1-5). Irrespective of antimicrobial treatment, patients improved within 1 to 2 days after their GP consultation. When comparing the speed of improvement of the patients who did and did not take antimicrobial therapy, no difference was observed in reaching a score 2 or less on the pain scale. This was similar for reaching a score of 3 or less (Cox proportional hazards not significant).

**Table 5:** Change in the severity of symptoms of the UTI diary app patients from day 1 to day 2.

Severity of symptoms	No antimicrobial prescription (n=13)	Antimicrobial prescription (n=33)	Total (n=46)
Worse	4 (31)	21 (64)	25 (54)
Same	4 (31)	8 (24)	12 (26)
Better	5 (39)	4 (12)	9 (20)

**Table 6:** The severity of symptoms rated by UTI diary app users over 5 days.

Prescribed antimicrobial therapy	Daily pain scale score, n																									
	Day 1					Day 2					Day 3					Day 4						Day 5				
	1	2	3	4	5	1	2	3	4	5	1	2	3	4	5	1	2	3	4	5	1		2	3	4	5
No	7	3	4	2		4	2	3			5	1	1			2		1	1		1		1			1
Yes	14	15	8	7	4	17	3	5		1	12	3	1	2		7		2			9					1

## Discussion

This study shows the feasibility of collecting real-time data through novel mobile data collection methods, such as text messaging and mobile phone apps. These methods have the advantage of collecting data in real time across multiple time points. The respondents of this research were predominantly female, which is reflective of the profile of a UTI patient.

### Uptake by Patients

There was little variation in response between text message survey, UTI diary, and online survey, but more patients participated in the text message survey than the UTI diary app. There may be a number of reasons for this observation. Firstly, the profile of the UTI diary app users was younger compared to text message participants. Because the mean age of patients within the SIMPle study was 56.1 (SD 20.7) years [14], this age group may favor text messaging over mobile phone app. Secondly, the researchers were reliant on the GP to obtain patient mobile phone numbers. Post intervention interviews with GPs who participated in SIMPle indicated that some GPs found it difficult to explain to patients why they were requesting their mobile phone numbers, some GPs just forgot to ask patients, whereas others choose not to ask some patients (ie, elderly patients) thereby introducing selection bias. Therefore, not every patient was asked to participate. Thirdly, there was also a delay of one week between the launch of the SIMPle intervention and the availability of the UTI diary app, which meant that many GPs did not receive a demonstration of the UTI diary app. Improving the uptake of any mobile phone app for use in general practice requires full collaboration from the GP to be able to encourage download. Our study missed the buy-in of all GPs due to the delay.

### Text Message Design

A total of 351 patients opted in to the text message survey process, which represented a response of 37%. Each text message needed to be 160 characters or less, the researchers were restricted in what they could ask and how questions were presented. This meant that it was difficult to ask validated questions, particularly because each text message was required to contain opt-out instructions.

Questions were organized to follow the natural resolution of UTI and a 24-hour delay was implemented between each question in our survey to allow the resolution of symptoms before the final question. This may not have been clear to participants because some responded a few times to the same questions. An automated thank you message and better communication about the structure of the survey can improve this.

Repeating the question in the case of no response reminded participants to complete the entire series of questions. This strategy seemed to work well and is recommended if text messaging is considered.

### Mobile Phone App Design

The design of the UTI diary app allowed the researcher to capture real-time information on the participant and their symptoms over 7 days. Unlike the text message survey, its design was not restricted by character length; however, cost of design may be an issue.

Reminder messages (push notifications) were built into the UTI diary app, but it is unclear if these were helpful for the participants or whether participants turned these off manually.

The potential richness of data available through the UTI diary app was also an important factor when designing this app. The findings from the UTI diary app identified differences in prospective and retrospective reporting of severity of symptoms. Patients recalling severity of symptoms retrospectively (via online survey) were more likely to rate them as severe compared to those who were asked to rate symptoms in real time (via UTI diary app).

Similarly, this feasibility study showed little or no association between type and severity of symptoms and antimicrobial treatment because the majority of patients received an antimicrobial prescription (UTI diary app: 72%, 51/71; online survey: 95%, 86/91). Most patients seem to visit their GP around the peak of symptom severity. Irrespective of treatment, most patients improved within one or at the most two days. This seems to suggest that symptoms improve before the antimicrobial treatment can have an effect, which is suggested to take 24 to 48 hours. Although the sample size is too small to draw conclusions from this data, it highlights avenues for further research. These issues should be further examined in the randomized controlled trial (RCT) setting where the combination of the UTI diary app within an RCT comparing antimicrobial and symptomatic treatment will provide further insight.

### Data Analysis

All data were automatically uploaded to an encrypted server that the researcher could access. This made the analysis process more efficient and because data were received in real time the researchers could observe the uptake of the various data collection methods.

To our knowledge, no other studies have captured data on patient symptoms, treatment, and duration of symptoms using a text message survey or mobile phone app. The UTI diary app captured data in real time allowing researchers to track the progression of a UTI from consultation to when participants were symptom-free.

Data presented in this feasibility study are limited and results should guide further research. However, even though sample size was limited, the results are intuitive; real-time data can be used to capture a greater understanding of actual severity and symptoms compared to other methods. The impact of prescribing antimicrobial therapy on the duration or severity of symptoms could not be established due to the small sample size, but the results may indicate that antimicrobial treatment is not always necessary.

Participants could turn off reminder messages resulting in incomplete diary entries. However, this feasibility study showed that the collection of patient data through mobile phone apps is feasible and highly effective to collect real-time data on the natural course of the infection, subject to treatment. Participants should be made aware of the importance of daily entries when downloading the app and this should be part of the education for both the GP and patient.

### Conclusion

Due to the response rate associated with the UTI mobile phone app within this feasibility study, it is difficult for the authors to conclusively outline how text messaging or mobile phone apps can help improve patient outcomes. However, this feasibility study does identify the potential for bridging the gap between data collection from patients recruited from multiple research sites in clinical studies and disseminating the results to improve clinical practice. This feasibility study highlights that when a patient begins to engage with a data collection method related to their illness, in this case text messaging or a mobile phone app, they are likely to continue to do so in the end. By collecting patient data in real time through mobile methods, this study highlights the potential of monitoring the symptoms of patients with acute, short-lived illnesses, data that have been difficult to capture in the past due to minimal interaction with the patient after their initial consultation. This knowledge highlights the potential of capturing patient symptom data in real time in the future within the clinical setting, with the possibility of opening up a dialog between patients and GPs.

Retrospective accounts of illnesses are often used in primary care to diagnose illnesses. There are no studies to our knowledge that report real-time versus retrospective reporting of symptom type and severity for UTI. A study comparing real-time reporting of schizophrenic patients used mobile devices to provide real-time data on their symptoms for 7 days. The same patients were then asked to complete a survey. Their results showed that retrospective accounts through surveys captured average ratings only and surveys were unable to capture variability of symptoms over time [15]. This feasibility study showed similar trends; however, more research is needed to investigate this further.

Symptom diaries for lower respiratory tract infections have been shown to be easy to use for measuring symptoms and treatment effects [16]. Diaries have also been used in the past to investigate natural course and treatment options for UTI. In Little et al’s study [17], only 64% of participants returned complete symptom diaries. The bias of incomplete data could be avoided with an app that electronically extracts data entries each day. In another study on antimicrobial use in UTI, researchers used follow-up telephone calls three days after initiation of treatment to remind patients to complete their diaries. Patients also received a follow-up call 28 days after their initial consultation to remind them to complete the survey and return a urine sample [18]. These methods are labor intensive and biased due to retrospective recording of symptoms and treatment remains an issue.

Within a recent RCT comparing antimicrobial therapy with symptomatic treatment, a diary was used to measure severity of symptoms and treatment compliance [19]. However, to maximize data collection and quality, they used study nurses to make telephone calls at days 1, 3, 5, and 7 to record symptoms and treatment [20]. Even though it improved data quality, this cost can potentially be saved with the use of the UTI diary app, in which reminders and pop-ups can help patients record their symptoms and treatment.

It has been shown that electronic diaries (palm-held devices) with enhanced compliance features were a more effective method of collecting information in comparison with paper-based diaries for chronic pain [21]. This study showed that compliance with paper-based diaries was poor compared to electronic diaries; patients did not complete their paper-based diaries in a timely fashion (ie, backfilling diaries) introducing bias due to retrospective recall and systematic bias because of the self-selection of completion times. To reduce any retrospective recall bias, diaries should be completed close to the time of the event they are trying to measure (ie, antibiotic consumption) [22].

The ubiquitous use of mobile phones provides opportunities to collect high-quality, real-time data through easy-to-use apps. Paper-based surveys can also be cumbersome and inconvenient to access depending on their design. Apps have been shown to be acceptable for patients and to save time and money in health research.

## Abbreviations

GP: general practitioner
RCT: randomized controlled trial
SIMPle: Supporting the Improvement and Management of UTIs
SMS: short message service
UTI: urinary tract infection
